# Modeling seasonal emergence of *Poa annua* in urban greenspace

**DOI:** 10.1038/s41598-021-98525-4

**Published:** 2021-09-23

**Authors:** Dallas R. Taylor, Michael Prorock, Brandon J. Horvath, James T. Brosnan

**Affiliations:** 1 Department of Plant Sciences, The University of Tennessee, Knoxville, TN USA; 2 Mesur.io., Yanceyville, NC USA

**Keywords:** Plant development, Plant sciences, Environmental sciences

## Abstract

Turfgrasses are perennial components of urban greenspaces found in parks, recreational areas, golf courses, sports fields, and lawns that confer many ecosystem services. A copious seed producer, *Poa annua* is the most troublesome weed of turfgrass and continually threatens the ecosystem services provided by urban greenspaces. Field research was conducted in Knoxville, TN to better understand environmental conditions triggering *P. annua* seedling emergence patterns to assist managers with optimally timing interventions—both chemical and non-chemical—for control. Fluctuations in cooling degree day (CDD_21C_) accumulation accounted for 82% of the variance in yearly cumulative *P. annua* emergence data collected in a single irrigated sward of hybrid bermudagrass [*C. dactylon* (L.) Pers. x. *C. transvaalensis* Burtt-Davy]. However, non-linear models using CDD_21C_ data developed *ex post* were not able to accurately predict *P. annua* emergence patterns *ex ante*. In both years, *P. annua* emergence changed most rapidly between the 40th and 43rd week of the year when seven-day mean soil temperature and rainfall were 18.9 °C and 12.7 mm, respectively. Future research should explore the efficacy of herbicide mixtures applied when *P. annua* emergence is most rapidly changing in lieu of developing models to predict when specific emergence thresholds occur.

## Introduction

Urbanization of the world’s population has increased focus on urban greenspaces and the ecosystem services they provide to people living in cities. Established sustainability goals set forth by the United Nations and the United States center upon mitigating increases in global temperature and protecting biodiversity to conserve ecosystem services^1,2^. Turfgrasses are perennial components of urban greenspaces found in parks, recreational areas, golf courses, sports fields, and lawns that confer a multitude of environmental and societal benefits from atmospheric enhancement to improved mental and physical well-being that have been reviewed in the literature^3^. *Poa annua* (annual bluegrass) is the most troublesome weed of managed turfgrass in urban greenspaces^4^. Having been documented on all continents including Antarctica^5^, *P. annua* is highly adaptable and noted for having ecotypes varying in morphological characteristics^6^. A day-neutral flowering plant^7^, *P. annua* seed germination has been studied in growth chamber environments with ecotypes germinating independent of daylight and across an air temperature range of 10 to 39 °C^8^. At one location in Maryland, multiple *P. annua* seedling emergence events were noted in autumn, with peak emergence (50 to 70%) occurring during a four-week period when air temperatures measured ≤ 20 °C^9^. While information about air temperature effects on *P. annua* emergence is useful, a better understanding of factors such as soil temperature and soil moisture is warranted, particularly considering this species environmental adaptability. Additional information about edaphic factors triggering *P. annua* emergence could help managers combat widespread herbicide resistance in this species^10^ given that soil seedbank management is noted as a best management practice (BMP) for combatting resistance^11^.

Models have been developed using climatic data to aid managers in making pest management decisions in turfgrass systems. Heat accumulation, expressed as growing degree days (GDD), has been used to increase efficacy of plant growth regulator applications to both cool- and warm-season turfgrasses^12–15^ as well as to schedule preemergence herbicide applications for smooth crabgrass (*Digitaria ischaemum* Schreb.) control^16^. Recently, a model that tracked a five-day moving average of daily relative humidity and daily average air temperature was developed to aid turfgrass managers in making fungicide applications to control dollar spot (*Clarireedia homoeocarpa*)^17^.

Ecosystem services provided by urban greenspaces are continually threatened by *P. annua* given that the species produces upwards of 100,000 seeds per square meter^18–20^. There is also widespread evidence that once established, *P. annua* can persist perennially^6^. There is a need to better understand environmental conditions triggering seedling emergence patterns to assist turfgrass managers with optimally timing interventions—both chemical and non-chemical—for *P. annua* control. The objective of this research was to model the emergence of *P. annua* in hybrid bermudagrass [*C. dactylon* (L.) Pers. x *C. transvaalensis* Burtt Davy].

## Materials and methods

Our research involved the use of plant materials and all required permits and permissions required to conduct studies were obtained prior to trial initiation. All experiments were conducted in accordance with institutional, national, and international guidelines and legislation.

### Yearly cumulative emergence

Field research was conducted at the East Tennessee AgResearch and Education Center- Plant Sciences Unit (ETREC; Knoxville, TN; 35.90°N, − 83.95°W) from January 2019 through December 2020. The research site was located 255 m above sea level. *Poa annua* emergence was monitored in hybrid bermudagrass (cv. ‘Tifway’) maintained at 1.5 cm with a reel mower during periods of active growth; the site was not mowed during winter dormancy. This research site had a natural history of herbicide-susceptible *P. annua* infestation. Soil was a Sequatchie silt loam (fine-loamy, siliceous, semiactive, thermic humic Hapludult) with soil pH 6.2 and water pH 6.1. Phosphorus (Mehlich-I), potassium, calcium, and magnesium concentrations in soil measured 11, 73, 970, and 106 ppm, respectively.

Plots (1 m × 1 m) were arranged in randomized complete block design (RCBD) with four replications. Within each replication, one plot was maintained as bare soil while the other was maintained as a hybrid bermudagrass. These two conditions were chosen to facilitate collection of emergence data over the widest span of turfgrass canopy cover. Bare-soil plots were maintained by physically removing aboveground hybrid bermudagrass biomass via scalping prior to the start of the experiment and periodically applying glyphosate at 1120 g ha^−1^ (Roundup Pro. Bayer Environmental Sciences. St. Louis, MO) after collecting *P. annua* emergence data. Emergence was monitored inside a circle (1000 cm^2^) in the center of all eight plots. When an emerged *P. annua* seedling was present inside the circle, it was recorded, and then discarded with tweezers. Emerged weeds other than *P. annua* were removed using the same equipment and discarded. Each year *P. annua* emergence was assessed on a weekly basis from January 1 through May 31, biweekly from June 1 through August 31, and weekly from September 1 to December 31.

Environmental monitoring devices (Earthstream. Mesur.io. Yanceyville, NC) were installed in each plot to collect soil temperature (5 cm depth), air temperature, soil moisture (5 cm depth), and daily light integral data on 15-min intervals for the duration of the experiment. Meteorological data within a 20-m radius of plots were also collected from publicly available sources including the National Oceanic & Atmospheric Administration (NOAA), National Climate Reference Network, and the European Space Agency (ESA). Using data from auxiliary sources along with those captured by in-ground sensors reduced the risk of point sample information not accurately representing actual variable averages such that inferences could be made about *P. annua* across a wider scale than the immediate area around the in-ground sensor^21^.

### Auxiliary locations

In order to expand inference beyond ETREC, data were collected at auxiliary locations during the autumn of 2020: Lambert Acres Golf Course (Alcoa, TN; 35.75°N, − 83.88°W) and Three Ridges Golf Course (Knoxville, TN; 36.09°N, − 83.84°W). At each auxiliary location, *P. annua* emergence was monitored in bermudagrass maintained at a 3.8 cm height of cut. Two plots (1 m × 1 m) were placed at each location and separated ≥ 75 m from one another. Soil at Lambert Acres was an Emory silt loam (Fine-silty, siliceous, active, thermic Fluventic Humic Dystrudepts) with soil pH 5.4 and water pH 7.6. Phosphorus (Mehlich-I), potassium, calcium, and magnesium concentrations in soil measured 114, 250, 1595, and 148 ppm, respectively. Soil at Three Ridges was an Urban land-Udorthents complex with soil pH 5.9 and water pH 7.7. Phosphorus (Mehlich-I), potassium, calcium, and magnesium concentrations in soil measured 17, 278, 2150, and 230 ppm, respectively. Emergence of *P. annua* was monitored inside a circle (1000 cm^2^) in the center of each plot using methods previously described on a weekly basis from August 1 through November 30, 2020.

### Model development

Emergence data were fit to non-linear functions using the Python (Python 3.8.7; python.org 2020) programming language. Emergence was modeled as a function of cooling degree day (CDD_21C_) accumulation from the summer solstice given that Pearson’s correlation analysis found it to be the strongest predictor of changes in *P. annua* emergence *post-hoc* in 2019 (r = 0.90) of all 25 environmental parameters analyzed.

Several libraries were used in the model development process including ‘pandas’ for data handling (v. 1.2.0; https://pandas.pydata.org/), ‘NumPy’ for numerical operations (v. 1.19.0; https://numpy.org/), ‘scikit learn’ for model building (v. 0.24; https://scikit-learn.org/stable/), and ‘SciPy’ for curve fitting (v. 1.5.4; https://www.scipy.org/). Model visualization was conducted using both ‘matplotlib’ (v. 3.3.3; https://matplotlib.org/3.3.3/index.html) and ‘seaborn’ (v. 0.11.1; https://seaborn.pydata.org/).

#### Early season emergence

To better understand factors triggering *P. annua* emergence in autumn, early-season emergence (0 to 50% yearly total) and cooling degree day accumulation data were fit to the following Gompertz function [Eq. (1)] where,

1 $$ESE = a*e^{{ - b*e^{{ - c*{\text{CDD}}21{\text{C}}}} }}$$

ESE = early-season *P. annua* emergence (*%*), a = the upper asymptote of the emergence curve, b = the displacement of the emergence curve along the x-axis, c = the growth rate of the emergence curve, and CDD_21C_ = cooling degree days accumulated from the summer solstice using a 21 °C base temperature. This model was developed *ex post* using data collected from ETREC in 2019 and validated at three locations in 2020: ETREC, Lambert Acres Golf Course, and Three Ridges Golf Course.

#### Yearly cumulative emergence

To better understand the entirety of *P. annua* emergence throughout autumn, yearly cumulative emergence data were regressed against CDD_21C_ accumulation and fit to a ruminal degradation curve [Eq. (2)] where,

2 $${\text{YCE }} = \, - {\text{a }} + {\text{ b }}* \, \left( {{1} - e^{{ - {\text{c}}*{\text{CDD21C}}}} } \right)$$

YCE = yearly cumulative *P. annua* emergence (*%*), a + b = the y-axis offset of the emergence curve, c = the growth rate of the emergence curve, and CDD_21C_ = cooling degree days accumulated from the summer solstice using a 21 °C base temperature. This model was developed using data collected from ETREC in 2019 and validated using data collected there in 2020.

#### Model fit

In all cases, model fit was determined using three parameters: coefficient of determination (R^2^), mean absolute error (MAE), and mean squared log error (MSLE). MAE provided an assessment of error between paired observations expressing the same phenomenon^22^, while MSLE measured the average of the squares of these errors^23^. All statistical analyses are available in a Google collaborative environment at: https://colab.research.google.com/drive/1-FRzYuPFtLk-2u_26vzFcmW9_Zc_sEvi?usp=sharing.

## Results

### Early season emergence

**Figure 1:**
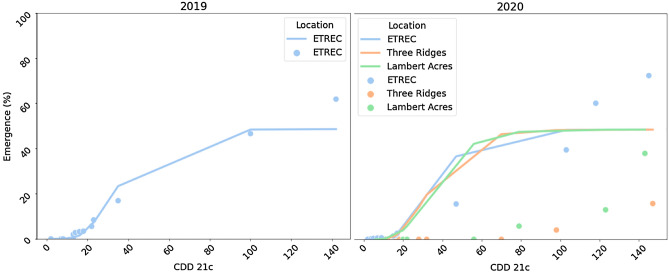
Gompertz function fit to early season (0 to 50% yearly maximum) *P. annua* emergence data collected at the East Tennessee AgResearch & Education Center—Plant Sciences Unit (Knoxville, TN) in 2019 and 2020, as well as Lambert Acres Golf Course (Alcoa, TN) and Three Ridges Golf Course (Knoxville, TN) in 2020. Emergence data were collected weekly with observations were regressed over cooling degree days (CDD_21C_) with accumulation beginning at the summer solstice using a 21 °C base temperature. Colored circles represent actual observations whereas the solid line represents model predictions. Measures of model fit for each location and year are presented in Table 1.

A Gompertz function fit early-season *P. annua* emergence data collected at ETREC well in 2019 (Table 1, Fig. 1). However, MAE and MSLE values for this model increased in 2020 at ETREC and the auxiliary locations. Moreover, R^2^ values were markedly lower in 2020 compared to 2019. For example, using data collected at ETREC, the R^2^ value for this model measured 0.74 in 2019 compared to 0.71 in 2020; values for the auxiliary locations in 2020 were ≤ 0.31 suggesting that this model fit emergence data at these sites worse than ETREC.

**Table 1:** Gompertz function to fit early season (0 to 50% yearly maximum) *P. annua* emergence and cooling degree day accumulation data collected at the East Tennessee AgResearch and Education Center—Plant Sciences Unit (ETREC; Knoxville, TN) in 2019 and 2020, as well as Lambert Acres Golf Course (Alcoa, TN) and Three Ridges Golf Course (Knoxville, TN) in 2020.

Year^a^	Location	R^2b^	MAE	MSLE
2019	ETREC	0.74	0.014	0.00081
2020	ETREC	0.71	0.021	0.00118
	Three ridges	0.26	0.037	0.00210
	Lambert acres	0.31	0.026	0.00145
	All locations combined	0.53	0.027	0.00154

Emergence was monitored in plots (1 m^2^) established as a hybrid bermudagrass (*C. dactylon* L. Pers*.* x *C. transvaalensis* Burtt-Davy) on a weekly basis.

^a^Early season *P. annua* emergence data and cooling degree days (CDD_21C_) accumulation data were fit to the following Gompertz function each year: Emergence (%) = 0.485 × exp (− 12.141 × exp(− 0.081× CDD_21C_)).

^b^Model fit was assessed using coefficient of determination (R^2^), mean absolute error (MAE), and mean squared log error.

At ETREC in 2019, *P. annua* emergence was documented when 12 CDD_21C_ accumulated (September 4). In 2020, initial emergence was documented at ETREC when 8 CDD_21C_ accumulated (August 5), almost a full month earlier than 2019. Earlier *P. annua* emergence could be related to cooler air temperature as monthly averages were lower in 2020 for both August (24.9 °C versus 25.8 °C) and September (21.2 °C versus 25.8 °C). Air temperatures where *P. annua* emergence was initially observed each year were within the 10 to 39 °C range previously reported in a growth chamber^8^. However, it should be noted that air temperature might not be the best predictor given that emergence was not documented at either auxiliary location in 2020 until 72 CDD_21C_ accumulated on October 1 (Fig. 1). This discrepancy in emergence timing between ETREC and the auxiliary locations may be related to soil moisture considering that plots at the auxiliary locations were irrigated only via rainfall whereas those at ETREC received both rainfall and supplemental irrigation. In a field study of cover crops, solarization, and fumigation on management weed seedbanks, *P. annua* emerged following rainfall events in late summer or early fall^24^. In our experiment with turfgrass, *P. annua* emergence occurred two days after auxiliary locations received 78.6 to 87.7 mm rainfall in a six-day period.

### Yearly cumulative emergence

**Figure 2:**
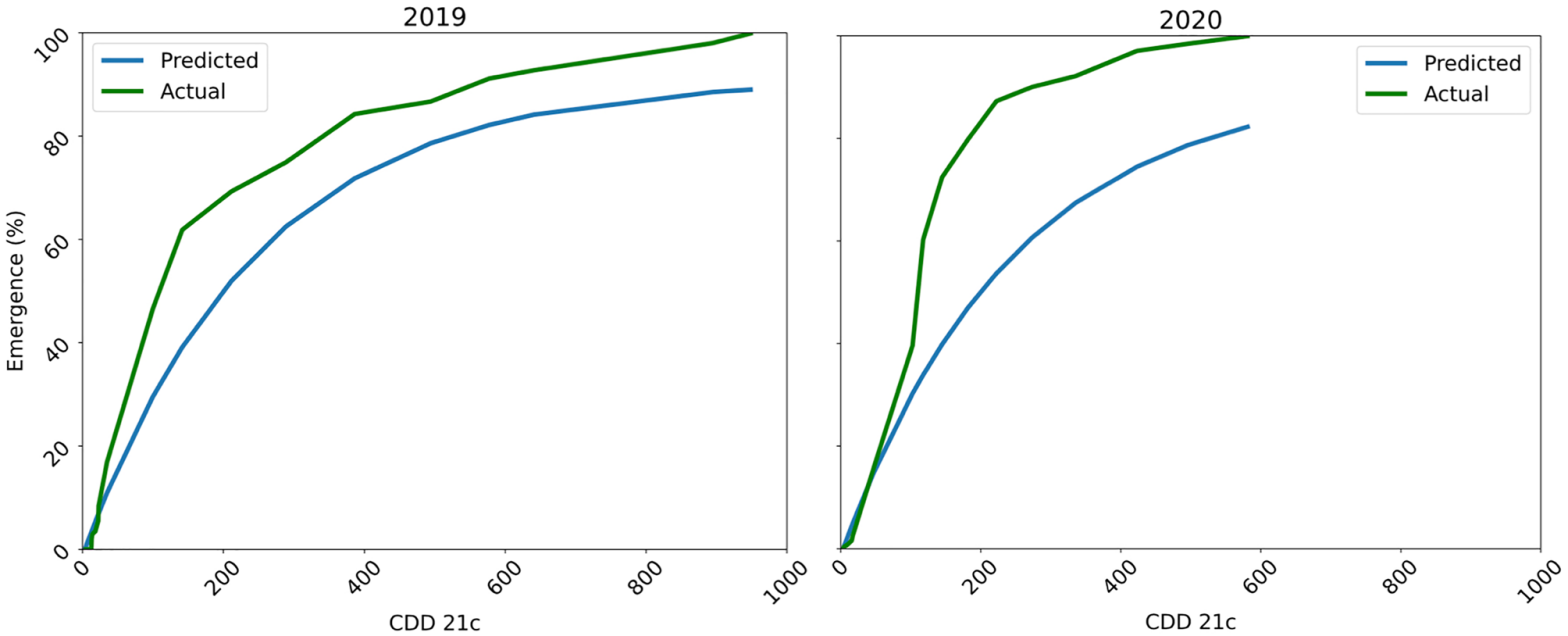
Four-parameter ruminal degradation curve to fit yearly cumulative *P. annua* emergence data collected at East Tennessee AgResearch and Education Center—Plant Sciences Unit (Knoxville, TN) in 2019 and 2020. Emergence was monitored on a weekly basis in plots (1 m^2^) established as a hybrid bermudagrass (*C. dactylon* L. Pers*.* x *C. transvaalensis* Burtt-Davy, cv. ‘Tifway’) fairway and maintained as bare soil. Combined observations were regressed over cooling degree day accumulation from the summer solstice using a 21 °C base temperature (CDD_21C_). The blue line represents model predictions whereas the green line represents actual observations each year. Measures of model fit for each year are presented in Table 2.

A ruminal degradation curve fit yearly cumulative *P. annua* emergence data collected at ETREC well in 2019 (Table 2, Fig. 2). However, MAE and MSLE values for this model increased in 2020 and the coefficient of determination decreased (Table 2). Despite the increases in overall MAE and MSLE values, findings illustrate that CDD_21C_ accumulation accounted for over 82% of the variation in *P. annua* emergence at the ETREC location over a 2-year period.

**Table 2:** Ruminal degradation curve fit to full season *P. annua* emergence and cooling degree day accumulation data collected at East Tennessee AgResearch and Education Center—Plant Sciences Unit (ETREC) (Knoxville, TN) in 2019 and 2020.

Year^a^	R^2b^	MAE	MSLE
2019	0.95	0.062	0.0031
2020	0.82	0.119	0.0111

Emergence was monitored on a weekly basis in plots (1 m^2^) established as a hybrid bermudagrass (*C. dactylon* L. Pers. x *C. transvaalensis* Burtt-Davy, cv. ‘Tifway’) fairway and maintained as bare soil.

^a^Yearly cumulative *P. annua* emergence data and cooling degree days (CDD_21C_) accumulation data were fit to the following ruminal degradation function: Emergence (%) = − 0.01275 + 0.9220 + (1 − exp(− 0.004 × CDD_21C_)).

^b^Model fit was assessed using coefficient of determination (R^2^), mean absolute error (MAE), and mean squared log error.

In 2019, *P. annua* emergence was first documented at ETREC when 12 CDD_21C_ accumulated and continued throughout autumn. Emergence measured 50% by the time 141 CDD_21C_ had accumulated (October 27), and reached 75% by the time 288 CDD_21C_ accumulated (November 9). Using 2019 total plant count data, peak emergence (61% of the yearly total) occurred during the 4-week period between October 11 and November 3, which corresponded to 32 to 211 CDD_21C_. Average day/night air temperatures during this period were 20.3/8.2 °C, respectively, whereas soil temperature averaged 16.6 °C. These observations in 2019 align with the findings of Kaminski and Dernoeden^9^ who recorded *P. annua* emergence in cool-season turfgrass once air temperatures fell to 20 °C. Additionally, McElroy et al.^8^ reported that the optimal air temperature for *P. annua* germination in a growth chamber occurred at 19 °C.

In 2020, *P. annua* emergence was first documented at ETREC after 8 CDD_21C_ accumulated on August 5th and continued throughout autumn. Emergence in 2020 reached 50% and 75% by the time 117 CDD_21C_ (October 9) and 188 CDD_21C_ accumulated (October 22nd), respectively. Peak *P. annua* emergence (61% of yearly total) occurred during the four-week period between September 24 and October 16, which corresponded to 46 to 145 CDD_21C_. Average day/night air temperatures during this period were 22.7/11.2 °C, respectively, whereas soil temperatures averaged 18.9 °C. This period of peak emergence at ETREC occurred earlier in 2020 than 2019 and air temperature alone does not explain this difference considering that day/night air temperature averages during this four-week period were higher in 2020 than 2019. Interestingly, there were substantial differences in rainfall between years that may explain the shift in the period of peak emergence. In 2019, rainfall totaled 139.7 mm for the months September and October, respectively, compared to 226.6 mm in 2020.

The yearly cumulative emergence model developed using 2019 data collected at ETREC underpredicted emergence in 2020 (Fig. 2). For example, this model suggested that 50% and 75% annual bluegrass emergence would occur at 225 and 445 CDD_21C_, respectively. In 2020, these benchmark targets were met at 117 and 181 CDD_21C_, respectively. Increased soil moisture may have influenced *P. annua* emergence in 2020. Although ETREC received irrigation to supplement rainfall, there was an 87 mm difference in rainfall accumulation during September through October 2020 compared to 2019. Further, CDD_21C_ only accounted for 82% of the variability in emergence data collected in 2020 compared to 95% in 2019 (Table 2). This difference in rainfall over years may explain this change and suggests that future models incorporate both parameters.

## Discussion

While non-linear models were able to fit both early-season and yearly cumulative *P. annua* emergence data *ex post* in 2019, they were not able to accurately predict the same phenomenon in 2020. Future research could be conducted using the 24-month dataset generated in this study to develop new models to predict *P. annua* emergence benchmarks. However, a model with applicability over a wide geographic range would require a vast amount of data for validation. Is such a research effort worthwhile given the environmental adaptability of *P. annua* and the array of edaphic factors that can affect weed seed emergence from soil?

**Figure 3:**
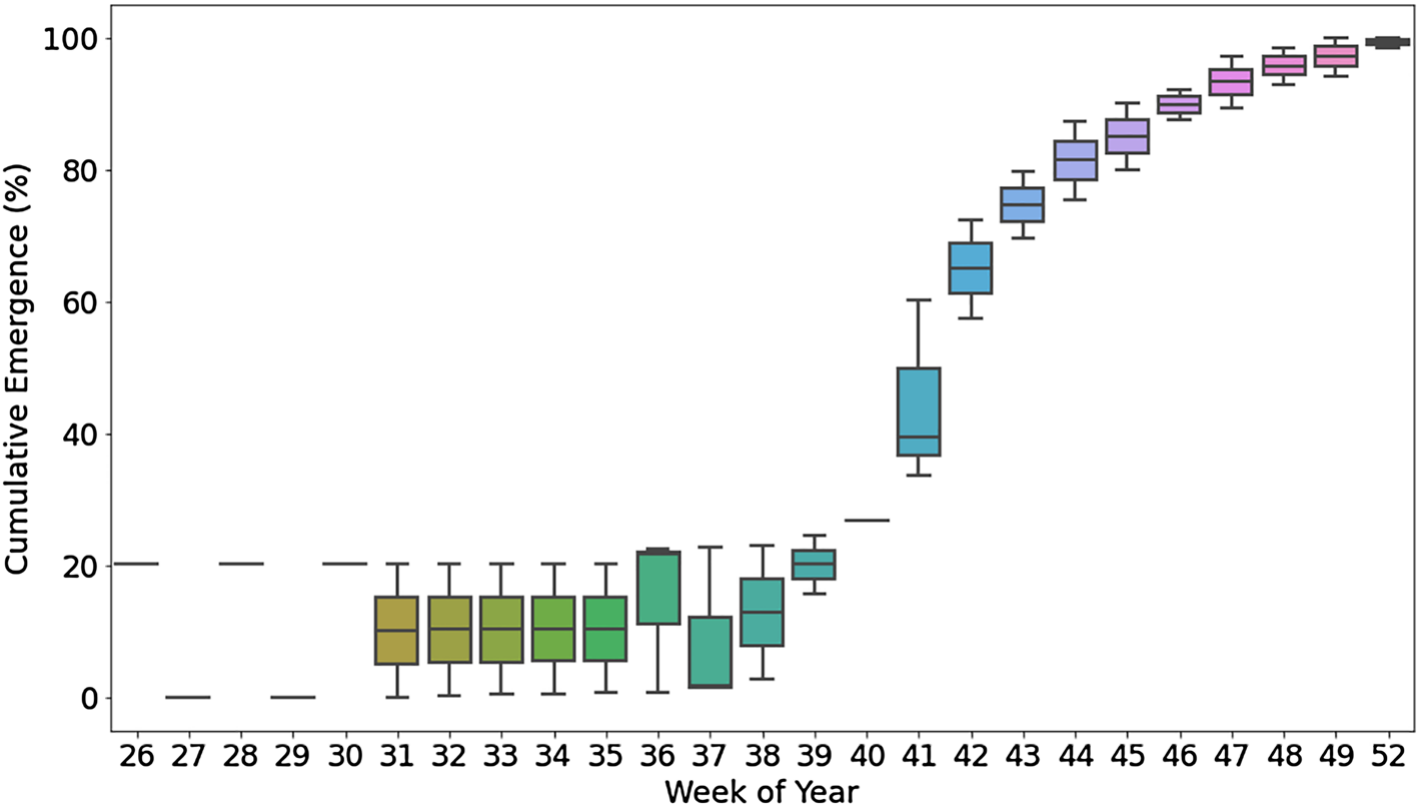
Weekly changes in *P. annua* emergence during 2019 and 2020 at three locations in East Tennessee: East Tennessee AgResearch & Education Center—Plant Sciences Unit (Knoxville, TN; 35.90°N, -83.95°W), Lambert Acres Golf Course (Alcoa, TN; 35.75°N, − 83.88°W), Three Ridges Golf Course (Knoxville, TN; 36.09°N, − 83.84°W). Boxes represent 25, 50, and 75% percentiles with minimum and maximum values represented by vertical bars.

Mixtures of pre- and postemergence herbicides are recommended for controlling weeds like *P. annua* known to evolve resistance to herbicides^25,26^. Managers are charged with applying pre- and postemergence mixtures when emergence is most rapidly changing. Cumulative *P. annua* emergence at all locations in this 24-month study is presented in Fig. 3. Emergence changed most rapidly between the 40th and 43rd week of the year. Interestingly edaphic factors during this window were consistent in both 2019 and 2020. Seven-day mean soil temperature (5 cm) was 18.9 °C (± 0.39 °C) and seven-day mean rainfall was 12.7 mm (± 0.6 mm). Future research exploring efficacy of pre- and postemergence herbicide mixtures applied for *P. annua* control using these benchmarks is warranted. Turfgrass managers often implement measures to control fungal pathogens such as *Rhizoctonia solani* AG2-2 LP and *Ophiospherella* spp. using similar edaphic benchmarks^27,28^ and may readily implement measures for *P. annua* control at this timing as well.

Overall, fluctuations in CDD_21C_ accounted for ≥ 82% of the variance in yearly cumulative *P. annua* emergence data collected in an irrigated hybrid bermudagrass fairway at ETREC. However, non-linear models developed *ex post* were not able to accurately predict *P. annua* emergence patterns *ex ante*. Given edaphic conditions facilitating the most rapid changes in *P. annua* emergence were similar over years, future research should explore efficacy of herbicide mixtures applied at this timing in lieu of developing models to predict when thresholds of *P. annua* emergence (e.g., 25%, 50%, etc.) occur.
